# The Oral Healthcare System in Japan

**DOI:** 10.3390/healthcare6030079

**Published:** 2018-07-10

**Authors:** Takashi Zaitsu, Tomoya Saito, Yoko Kawaguchi

**Affiliations:** Department of Oral Health Promotion, Graduate School of Medical and Dental Sciences, Tokyo Medical and Dental University, Tokyo 113-8510, Japan; zaitsu.ohp@tmd.ac.jp (T.Z.); saito.ohp@tmd.ac.jp (T.S.)

**Keywords:** oral health, healthcare system, dental workforce, public health insurance, oral health status, Japan

## Abstract

This paper describes the present Japanese oral healthcare system and outlines the future challenges and perspectives for Japan. Japan has developed a system for providing high-quality and appropriate health care efficiently through a universal health insurance system which has been in operation since 1961. This health insurance covers most restorative, prosthetic and oral surgery treatment. Therefore, all people can receive dental treatment at a relatively low cost, with the same fees applying throughout the nation. In Japan, public oral health services are provided by the local governments according to the life stage of their populations. These services are mainly conducted by private dental practitioners under contracts with local governments. National oral health data shows that the oral health of the Japanese population has improved over the last several decades. Future challenges and perspectives for Japanese dentistry include: tackling the regional differences in oral health, decreasing the cost of health expenditure, establishment of sustainable emergency oral healthcare services in times of disaster, and the development a new tele-dental system for remote areas without access to dental professionals.

## 1. Characteristics of Japan

Japan is located in Northeast Asia and is composed of four main islands and 6848 smaller islands. The land area is 378,000 km^2^ and the capital city is Tokyo [1]. Japan’s population is over 126 million and most Japanese reside in densely populated urban areas [2]. In 2016, the national gross domestic product (GDP) was 4.937 trillion United States dollars (US$) and it is the world’s third largest economy [3]. Health expenditure is around 10% of GDP. Japan has developed a system for providing high quality and appropriate healthcare efficiently in its communities through a universal health insurance system which has been in operation for more than 50 years. 

## 2. Dental Workforce

Three regulatory professional dental licenses are issued in Japan: dentists, dental hygienists, and dental technicians. For each profession, independent legislation exists: the “Dentists Act”, the “Dental Hygienists Act”, and the “Dental Technicians Act”. These acts describe and regulate the professions’ duties, roles, and ethics. There is no licensing system for dental chairside assistants. A survey of practicing healthcare professionals is conducted every two years by the Ministry of Health, Labor and Welfare (MHLW).

### 2.1. Dentists

In 2016, the total number of dentists was 104,533 [4]. The number of female dentists was 24,344, 23.3% of the dental workforce. The dentist ratio per 100,000 people is 82.4 practitioners, and, as in many nations, the distribution is unequal. The highest dentist to population ratio is in Tokyo (118.2), and the lowest is Fukui Prefecture (54.7); more than twice the regional difference of dentist distribution is observed. There are 68,730 dental facilities (mainly private dental clinics) in total throughout Japan.

Table 1 shows the number and proportion of dentists according to their roles or places of practice. More than 97% of the dentists (*n* = 101,551) engage in providing dental treatment at private or public dental institutions. The number of public dentists who engage in full-time administration work is only 348 (0.3%). Therefore, in Japan, most of the public dental activities are conducted by private dentists on a part-time basis. The “Dentists Act” describes the duties of dentist as follows: “*Dentists shall take charge of dental treatment, provide oral health guidance, and contribute to the improvement and the promotion of public health in order to secure a healthy life for the people*”.

For example, a local government municipality contracts with a private dental practitioner to carry out the role of a school dentist. Local government pays the contracting dentist as a school dentist, and the dentist is responsible for the performance of school oral health activities, usually in a part-time capacity. This public and private mixed dental performance is one of the unique characteristics of the Japanese oral healthcare system.

In Japan, there are 29 dental educational institutions: eleven national, one local governmental, and seventeen private universities. The total enrolment in the 29 dental schools in 2017 was 2720 [5]. Dental education is based on a model core curriculum. For quality assurance of the education conducted in each dental school, computer based tests (CBTs) and objective structured clinical examinations (OSCEs) are performed during the undergraduate course before dental students start clinical training. After six years of education, all students have to take a national board dental examination. The MHLW manages this national board examination and regulates the issuing of dental licenses. The pass rate of this national board examination is relatively low, around 65–70%. In 2018, 3159 dental students took the examination and 2039 passed (64.5%) [6].

Without passing this examination, a dental graduate cannot get a dental license. Further, after successfully getting a dental license, all new graduates participate in the compulsory residency clinical training program for more than one year. Following completion of a residency program, the graduate is free to choose the career path to follow as a dentist. Most prefer further study through postgraduate university courses, or to work at hospitals to improve their academic knowledge and technical skills for several years before entering private practice. 

### 2.2. Dental Hygienists

The number of active dental hygienists in Japan in 2016 was 123,831 [7]. The roles of dental hygienists are prevention of oral diseases, oral health education, and chairside treatment assistance. About 90% of dental hygienists (*n* = 112,211) work in largely private dental clinics, and about 5% (*n* = 6259) work in hospitals. The number of dental hygienists working in public sectors (i.e., prefectures, municipalities, and health centers) is 2754 (2.2%), and teaching staff in education institutes is 873 (0.7%). 

In total there are 166 dental hygienist education institutes. Most of these are 3-year-period vocational schools. Eleven schools however provide a 4-year-period university bachelor degree programs in the universities. Hygienists also need a national license, and the proportion of dental hygienists who pass the national examination is high and around 95%. Every year around 6500 new dental hygienists are produced. 

### 2.3. Dental Technicians

In 2016, the total number of active dental technicians was 34,640 [7]. Dental technicians make dental prostheses, based on dentists’ prescriptions. They are not allowed to take impressions directly from the patients. The number of dental technicians working in dental laboratory offices is 24,972 (72.1%) and working in hospitals or dental clinics is 9166 (26.5%). 

There are 54 dental technicians’ schools. Most of the schools provide 2-year-period education. Three universities have 4-year-period bachelor degree programs for dental technicians. After graduation, a pass in the national board examination is necessary to get a license to practice as a dental technician.

## 3. Public Health Insurance System in Japan

Japan is called a welfare country and public healthcare systems are well developed. Japan introduced a universal health insurance system for the entire population in 1961. It covers almost all medical and dental treatment and pharmacy care required by the population [8]. People can receive treatment at a relatively low cost, and the same fee is applied throughout the nation. In 2000, in response to the increasing aging of the population, Japan initiated a “long-term care insurance” to deliver health and welfare services for the elderly.

### 3.1. Health Insurance

Almost all practicing doctors and dentists are registered in the public national health insurance scheme as insured doctors, and provide treatment according to a fee-for-service system. In general, after receiving treatment by an insured doctor or dentist, patients pay 30% of the total cost to the clinic or hospital. The remaining 70% of the cost is paid to the clinical institutions by the insurance agency approximately two or three months later, based on the submitted fee claims. Therefore, the cost of insurance treatment provided is the same, throughout the nation, fixed by the fee schedule. There is no price difference between private and public institutions. 

There are certain exemptions. Low income earners do not necessarily have to pay the cost directly to the clinic. In addition, elderly persons may pay directly but at a reduced rate (10–20% of the cost) according to their income. Moreover, the Japanese health insurance system has a reimbursement scheme for patients who receive costly treatment services such as cardiac surgery, where the patient’s payment over a certain amount is refunded later. Under this health insurance system, Japanese people can receive high-quality health services at a relatively low cost, both in public and private institutions. The fee schedule is reviewed every two years and inclusions/exclusions of each treatment option within the insurance scheme is reviewed by an expert committee established through the MHLW.

Dental services under the national health insurance system are available for most restorative, prosthetic, and oral surgery treatment. They include services such as fillings, endodontic treatment, crowns, bridges, dentures, and extractions. Higher cost items (e.g., gold crowns and bridges, metal plate dentures, implants, and orthodontic treatment) are excluded. Preventive services are also excluded, as the current health insurance system only covers treatments for existing diseases. Delivery of dental treatment services to bed-ridden people at home or in aged care centers by dentists are also covered in this public health insurance scheme.

### 3.2. Long-Term Care Insurance 

To deal with the rapidly increasing aging population, in April 2000 Japan introduced the “long-term care insurance system”. This system provides various long-term care services in a comprehensive and uniform way for all eligible persons, so that they can lead independently as long as possible. The managing insurer of the long-term care insurance system is the municipality (local government), and the main eligibility criterion for those covered by the scheme is that they are aged 65 years or over. 

Based on the care plan established by a patient’s care manager, the patient contracts the service provider to make necessary arrangements so that the individual can use in-home care services or community-based preventive services. Facilities are also available for those in the aged care institutions. To use long-term care services, the long-term care insurance covers 90% of the service-related costs, while the remaining 10% of costs are paid by the user. 

The services provided under this scheme include home visit nursing, day-care or short-stay medical service, etc. In-home healthcare guidance, doctors, nurses, dentists, dental hygienists, or other medical professionals visit the homes of users who have difficulty in making a hospital visit and provide health maintenance instruction and care according to the patient’s medical and physical condition or environment.

After its launch, there was a rapid increase in the use of the long-term care scheme, especially the home care service. The long-term care insurance system has now come to have an important role as a system designed to assure an affordable and comfortable life for elderly people and their family members. 

## 4. Life Course Oral Healthcare System

According to each life stage of the population, many policies regulate the regional health services and describe the accountability of governments, related organizations and populations in Japan. Therefore, oral health services are provided as a part of the general health service, and the programs are based on the health related laws and acts (Table 2). 

In 2017, there were a total of 479 health centers throughout Japan. Among them, 363 centers were established in 47 prefectures, 93 centers in 74 designated cities, and 23 centers within the 23 special Tokyo wards. These health centers take the role of the central administrative management office for the regional public health services.

In 2000, a National Health Promotion Campaign for the 21st century, “Healthy Japan 21”, was proposed to prevent lifestyle-related diseases (non-communicable diseases (NCDs) such as cancers, cardiovascular diseases, diabetes, and chronic obstructive pulmonary disease). “Healthy Japan 21” set up national goals for the year 2010 in nine specific fields for improving lifestyles, reducing risk factors, and decreasing diseases. Oral health is one of the NCD conditions identified, and specific goals were set up to prevent tooth loss. The “Health Promotion Act” was enacted in 2003 and it supported the development of health promotion activities throughout the nation.

After evaluation of the achievements on “Healthy Japan 21”, the second term of “Healthy Japan 21” was initiated from 2013. Its basic goals were as follows:

- Extension of healthy life expectancy and reduction of health disparities
- Prevention of onset and progression of life-style related diseases
- Maintenance and improvement of mental and physical functions necessary for social life
- Establishment of a healthy and supportive social environment

Specific goals for the year 2022 are indicated in these six fields, and include; (1) nutrition and dietary habits; (2) physical activity and exercise; (3) rest; (4) alcohol use; (5) tobacco use; and (6) oral health. Table 3 shows the oral health goals set out in the second “Healthy Japan 21”.

### 4.1. Preschool Children

Pregnant women receive a “maternal and child health handbook” from the municipal government for each child. Health care professionals record the health check-up data during pregnancy and after the child is born and up to six years of age. The handbook covers the child’s health condition and immunization records. Mothers also record the child’s growth and health concerns in the handbook by themselves. Therefore, healthcare professionals in hospitals or health centers can refer to the records within this book, as mothers always carry this book with the child. 

In Japan, national programs for preschool children are conducted by local government free of charge. They include physical, medical, and dental examinations of all children. The collected data are sent to the MHLW and published every year.

(1) Health check-ups for 3-year-old children (since 1961)

(2) Health check-ups for 18-month-old children (since 1977)

Private practitioners (i.e., doctors and dentists) contribute to the conduct of these examinations in turns at the community health centers. This means they become part-time “public doctors/dentists”. Medical or dental treatment is not provided at the health centers and only preventive services are available. After the oral examination, oral health education is offered to mothers and children by dental hygienists, either in a small group or individually. Education covers oral health related habits, nutritional consultation, and brushing instructions. Topical fluoride application for caries prevention and silver diamine fluoride application for caries arrest is also provided to those who require this care, at a reasonable fee.

### 4.2. Schoolchildren

In Japan, every public primary, junior, and senior high school has an appointed school dentist. In 2014 the total number of school dentists holding such positions was 44,600. The school dentist is responsible for the performance of school-based oral health activities, usually in a part-time capacity, because s/he may work also as a dental practitioner in the area. 

The roles of school dentists are described in the “School Health and Safety Act” and include the conduct of an oral health examination at least once a year on each child at school, and contributing to implementing the school’s oral health education. According to the standard procedures and guidelines, school dentists check the oral health status of all the students for conditions such as dental caries, malocclusion, gingival status, dental plaque, and temporomandibular disorders.

If oral health problems are detected in schoolchildren, the school dentist recommends to the child and parents that they should seek dental treatment under the public health insurance scheme, described before. School dentists do not provide dental treatment in the schools at all. Schoolchildren can receive comprehensive dental care at any public or private dental offices. 

In addition, oral health education is conducted by the school dentist, or the dental hygienist, in cooperation with the nursing teachers and the classroom teachers. Oral health education programs usually include prevention of dental caries and gingivitis, but the content of oral health education program depends on the individual school’s curriculum and timetable.

School health surveys are conducted every year, and the data are published by the Ministry of Education.

### 4.3. Adulthood

According to the “Industrial Safety and Health Act”, employers have to provide annual medical check-ups for all the employees in any company which has more than 50 workers. On the other hand, the Act does not include a duty for dental check-ups for employees. Only the workers who engage in jobs in acid-producing environments have to receive special dental check-ups every six months for the prevention and early detection of tooth erosion. Some companies provide good oral health promotion programs for their employees, but the number of these companies is very small.

According to the “Health Promotion Law”, local governments (municipalities) are to provide free or low-cost “periodontal disease examination programs” for their adult population by way of contracts with private dental practitioners. However, the rate of participation for the eligible persons in these programs is very low, about 10–15%. 

Therefore, in Japan, the oral health program for the adult population is based on an individual’s personal responsibility for care, self-support and self-motivation. Many dental facilities and a public insurance system contribute to easy access for dental treatment for adults, but the proportion of regular (check-up or preventive) visits to dental clinics is not high. This adult population group should be encouraged to visit a dentist and dental clinic regularly for prevention of dental diseases.

### 4.4. Elderly

Japan is known as a “super aging society”. The age structure (2016) shows that 12.4% of the population is aged 0–14 years, 60.3% is aged between 15 and 64 years, and just over a quarter of the population, 27.3%, is aged 65 years and older [9]. Life expectancy at birth (2016) is 81.0 years for males, 87.1 years for females, and 84.2 years for all [10].

This figure shows that Japan is one of the longest life expectancy countries in the world. Therefore, over the past several decades, Japan has become increasingly concerned at the pace of population aging and the challenges this brings to dealing with changing social systems.

Dentistry is no exception. In 1989, the Ministry of Health and the Japan Dental Association advocated a national oral health campaign, “8020 (Eighty-Twenty) campaign”. The first part “80” signifies the average life expectancy for Japanese people at that time, and the second part “20” indicates the critical number of natural teeth to maintain eating and chewing function for life. Previous studies in Japan show that keeping 20 or more natural teeth is considered to be a simple and adequate threshold for maintaining good masticatory ability for eating almost any kinds of Japanese food items, which vary from soft texture food to hard texture food [11]. 

The objective of this campaign is to inform the general population of the importance of retaining 20 or more natural teeth until 80 years of age to maintain satisfactory masticatory abilities. The number of missing teeth increases as people get older. The concept of “8020” is to ensure all Japanese people are able to enjoy a healthy diet and a good social life by preventing tooth loss that leads to masticatory dysfunction.

This national campaign has led to many projects and research studies regarding the impact of oral health on general health and quality of life. Many studies report that improvements in oral health and masticatory function contribute to the prevention of aspiration pneumonia and to the maintenance or recovery of activities of daily living [12]. In March 2015, the Japan Dental Association hosted the world congress with co-sponsorship by the World Health Organization (WHO), and the “Tokyo Declaration on Dental Care and Oral Health for Healthy Longevity” was drafted [13].

The “8020” campaign, a community and clinic-based initiative started in 1989, has contributed to a dramatic improvement in the oral health of older people in Japan. This was followed by an accumulation of evidence, culminating in oral health being integrated into health policy in the form of the “Act on the Promotion of Dental and Oral Health” in 2011, for the purpose of oral disease prevention and general health improvement.

Oral functional impairments reduce chewing efficiency, influence nutritional deficiencies, and deter the elderly from the pleasure of eating and communication. Oral functional enhancement, along with dental prostheses and better oral hygiene has been reported to be effective in preventing swallowing difficulties in the dependent elderly. From the perspective of prevention and health promotion, it is considered to be more effective to implement interventions before health problems and functional disturbances have occurred. Therefore, at community health centers, dental professionals educate the independent elderly about the importance of oral function promotion and provide oral function promotion programs such as “tongue exercise” or “salivary grand massages”.

## 5. Oral Health Status

In Japan, national oral health surveys have been conducted every six years from 1957 to 2011 by the MHLW. Recently, the eleventh survey was conducted in 2016, the interval between surveys being changed from six to five years. According to data from these surveys, the changing patterns of oral health status of Japanese population can be well described.

### 5.1. Oral Health Status of Children

**Figure 1:**
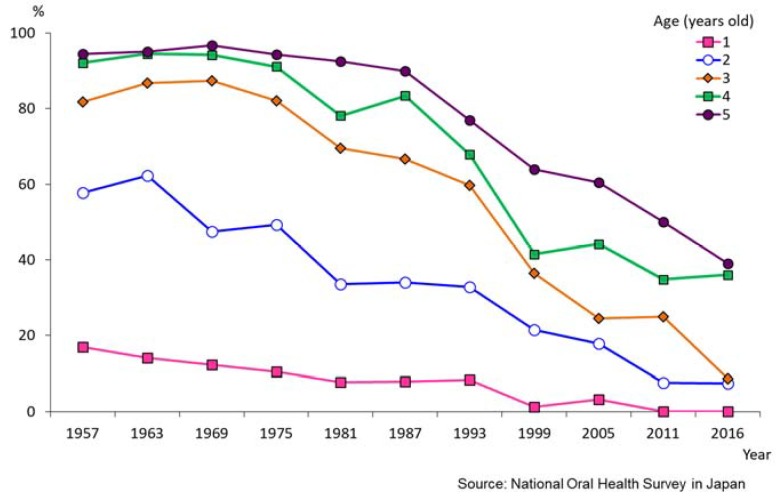
Trends in prevalence of dental caries, deciduous teeth (1957–2016) [15].

**Figure 2:**
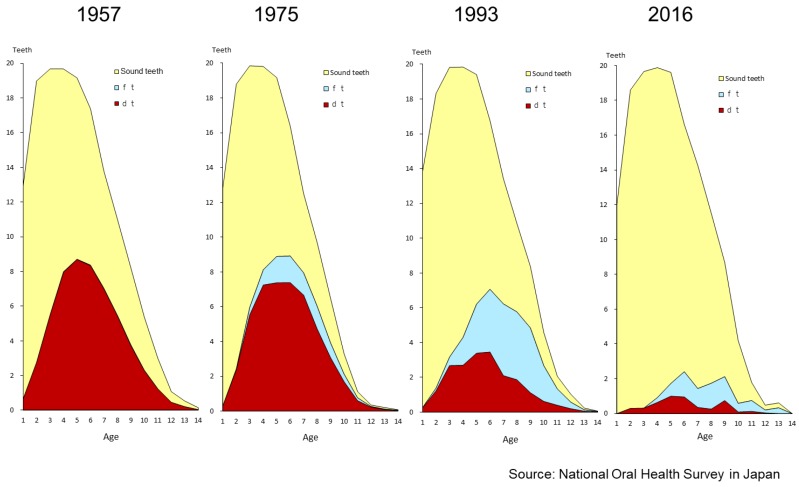
Changing pattern of deciduous teeth (1957–2016) [15].

**Figure 3:**
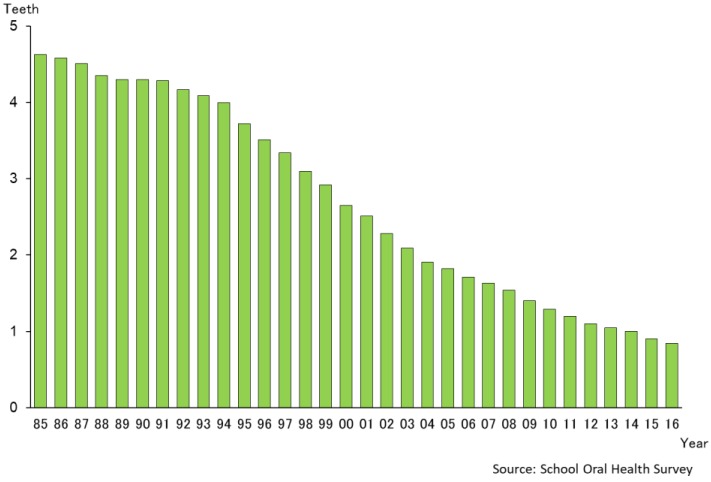
DMFT of in 12-year-olds (1985–2016) [14].

For deciduous teeth, improvement is obvious. Figure 1 shows the trends in prevalence of dental caries in deciduous teeth for one- to five-year-old children. In 1957, the prevalence of dental caries in 5-year-olds and 3-year olds were 94.5% and 81.8%, respectively. In 2016, these values decreased to 39.0% and 8.6%, respectively. Figure 2 shows the changing pattern of the status of deciduous teeth from 1957 to 2016 for one- to 14-years-old children. In 1957, most carious teeth were untreated, and 5-year-olds had on average 8.7 decayed teeth (dt). As time went on, children could access and receive dental treatment, and the number of filled teeth (ft) increased. Also the number of healthy teeth increased remarkably in all ages. These figures show the dental caries status of deciduous teeth in Japanese children improved rapidly. Figure 3 shows the changing pattern of decayed, missing and filled permanent teeth (DMFT) of 12-year-olds from national School Oral Health Survey data. In 1985, 12-year-olds had on average 4.6 DMFT, and this gradually decreased year by year and it became 0.8 DMFT in 2016 [14].

### 5.2. Oral Health Status of Adults

**Figure 4:**
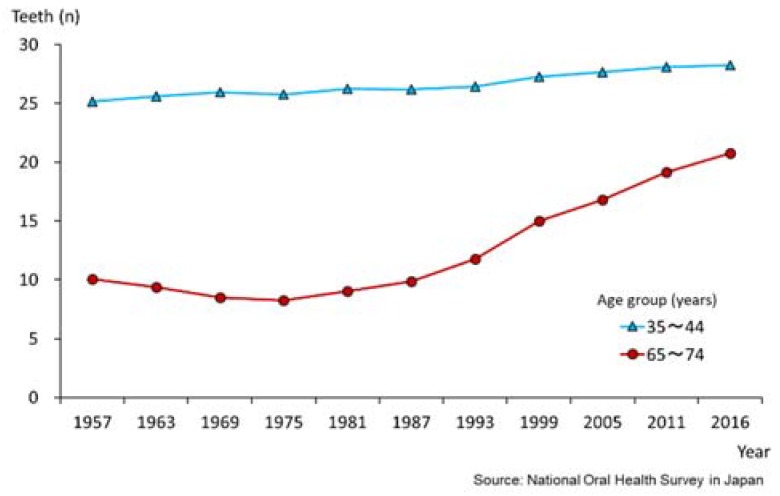
Changing pattern of mean number of present teeth for 35–44 and 65–74-year old groups (1957–2016) [15].

Figure 4 shows the mean number of teeth present for adults (35–44 years age group) and older persons (65–75 years age group) over a 60-year period from 1957 to 2016. For the 35–44-year age group, the number of natural teeth present increased from a mean of 25.1 to 28.2, a difference of more than three teeth. For the 65–74-year age group, the increase in the number of natural teeth was more remarkable, from 10.1 to 20.8 teeth. That is by a factor of ten teeth or twice the number of natural teeth present over this time period. This implies that recent Japanese populations, especially elderly people, are keeping more natural teeth than the past [15].

**Figure 5:**
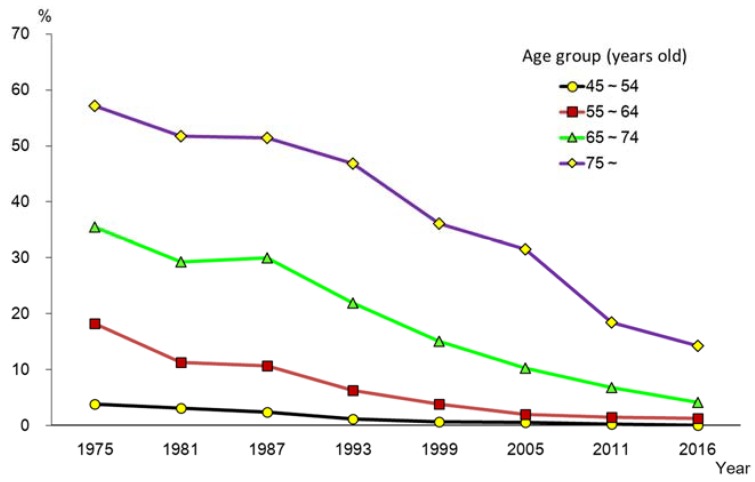
Trends in proportion of edentulous persons by age group (1975–2016) [15].

**Figure 6:**
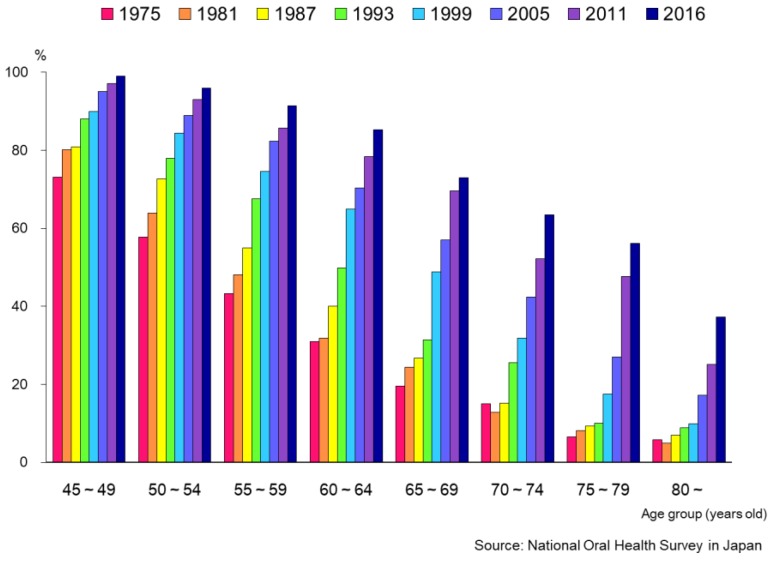
Proportions of persons with 20 or more teeth by age group [15].

On the other hand, the proportion of edentulous persons decreased each year in all age groups (Figure 5). In 1957, the proportion of those with no natural teeth was about one-third in the 65–74 year-old age group (35.5%), and more than half of those 75 years and over (57.2%). In 2016, these proportions had changed to 4.1% and 14.3%, respectively. Figure 6 shows the changing pattern of the proportion of persons with 20 or more teeth. In all age groups, the proportion of those retaining 20 or more natural teeth had increased, with a substantial increase observed, especially in older age groups. This might be attributed to the national “8020” campaign which was initiated in 1989, and people’s awareness for oral health which has been improving and changing oral health behaviors.

**Figure 7:**
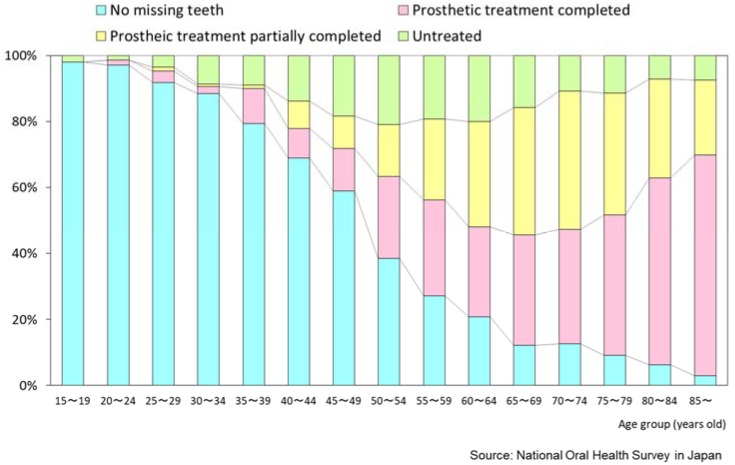
Prosthetic status (2016) [15].

Figure 7 shows the prosthetic status of those 15 years and over in the Japanese population in 2016. In total, the proportion without missing teeth (not needing prosthetic treatment) was 34.0%, and those who completed prosthetic treatment was 28.3%. In Japan, the public insurance covers most prosthetic treatments, such as dentures and bridges. Therefore, people can receive the prosthetic treatment they require also at a reasonable price.

**Figure 8:**
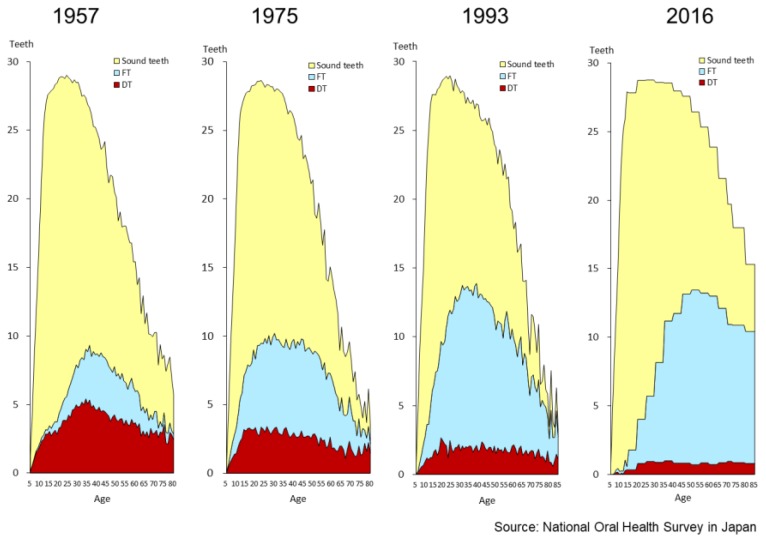
Changing patterns of permanent teeth (1957–2016) [15].

Figure 8 shows the changing pattern of the status of permanent teeth. In 1957, the number of decayed teeth was greater than the number of filled teeth in all age groups. In those days, the whole Japanese population was not covered by public health insurance. In 1961, all the population entered the public health insurance system and access to dental treatment improved. The number of decayed teeth on average decreased as time went on and the average number of decayed teeth (DT) was low at 0.8 teeth in the total population aged five years and over, in 2016. The number of healthy teeth in adults also decreased, and the number of filled teeth increased. Japanese health insurance is based on the fee-for-service system, so the more filled teeth, the more fees dentists can get. It is necessary therefore to consider the inclusion of prevention in the insurance schemes. As people keep more teeth than before, a preventive approach to dental care is more important.

### 5.3. Data on Oral Health Related Factors

Many factors are thought to be involved in the caries reduction of both deciduous teeth and permanent teeth in Japanese children. They include increased usage of different fluoride strategies, improvement of tooth brushing behavior, reduced sugar consumption as well as improved awareness of oral health through the public oral health check-up system for preschool and school children. 

**Figure 9:**
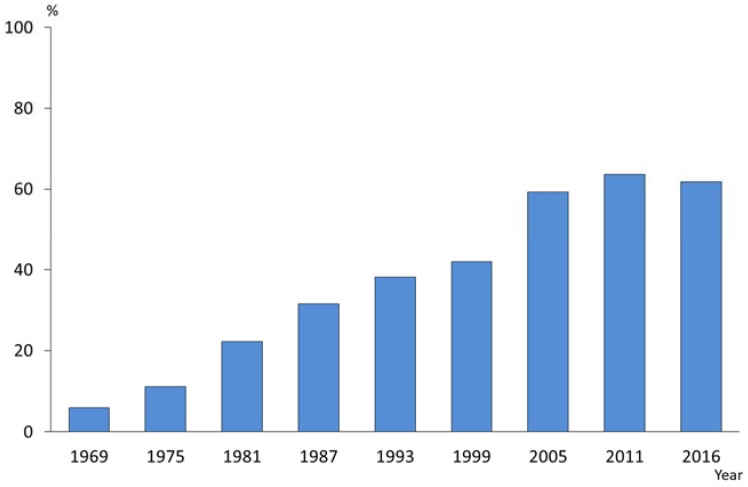
Proportions of persons (1–14 years of age) who had received topical fluoride application [15].

In Japan there is no systemic fluoride use, and only topical fluorides are available. Figure 9 shows the trends in the proportion of persons (1–14 years of age) who received topical fluoride application. In 1969, only 6% of children received topical fluoride application. Recent data shows that this increased to about 60% and indicated a 10 times increase in exposure [15].

**Figure 10:**
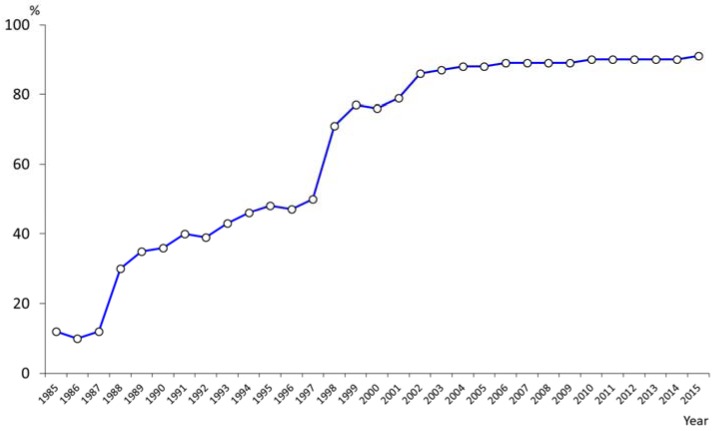
Market share of fluoride dentifrice (1985–2015).

**Figure 11:**
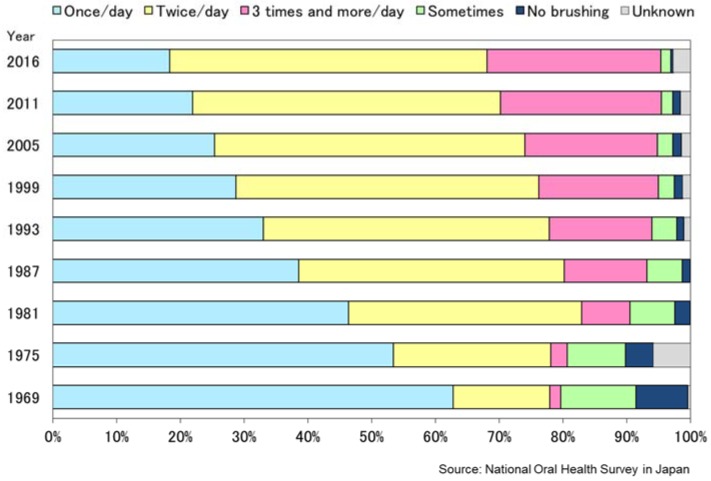
Reported tooth brushing habit (1969–2016) (1 year of age and over) [15].

**Figure 12:**
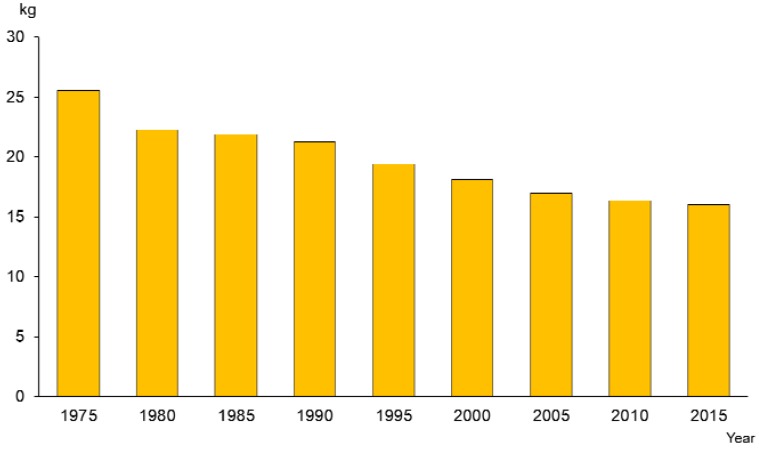
Sugar consumption per person per year [16].

The market share of fluoride toothpaste has also increased dramatically from 12% (1985) to 91% in 2015 (Figure 10). According to the National Oral Health Survey, tooth brushing behavior also improved for the whole population (Figure 11). Sugar consumption per person per year decreased from on average 27.5 kg per person in 1970 to16.1 kg in 2015, a difference of 11.4 kg (Figure 12) [16].

These factors, as well as the sufficient numbers in the dental workforce and the universal coverage of the public health insurance system have contributed to the improved oral health of all Japanese people.

## 6. Future Challenges for Japanese Dentistry

Although the oral health status of Japanese people has improved, there still remain many problems to be solved. These include: regional disparities in oral health and the total cost of health care, especially in the elderly. Further, as Japan is subject to many natural disasters, we have to establish an emergency oral healthcare system to cope in times of disaster and to train dental personnel to manage suitable intervention programs. It appears also important technologically to develop a new tele-dental system which can be used in the rural and remote areas of Japan without easy access to dental professionals to access diagnostic and preventive care—this is also one of our challenges for the future.

### 6.1. To Reduce the Regional Difference

**Figure 13:**
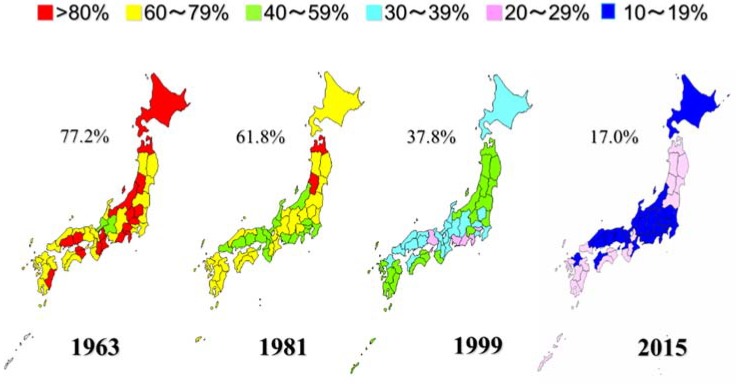
Regional differences of caries prevalence in 3-year-olds by year [17].

Japan consists of 47 prefectures. Figure 13 shows the regional differences in caries prevalence of three-year-olds according to the data from nationwide health examinations of three-year-old children. Caries prevalence in Japanese three-year-old decreased from 77.2% in 1963 to 17.0% in 2015 [17]. However, there remain substantial regional differences. In 2015, the caries prevalence in Okinawa prefecture (28.9%) was more than twice as high as Aich prefecture (11.2%). The number of carious deciduous teeth (dft) shows the same tendency. At present public dental services are offered based on the same rules and procedures throughout Japan. It might be advisable to develop special intensive preventive programs for high-risk persons or regions. 

### 6.2. To Decrease the Cost of Health Expenditure

**Figure 14:**
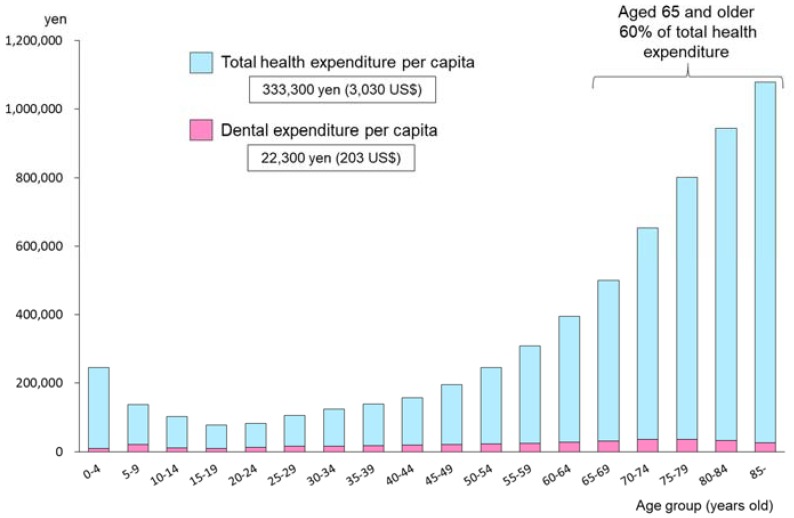
Total health expenditure and dental expenditure per capita by age group (Japan, 2015) (110 yen = 1 US$) [18].

Figure 14 shows the total health expenditure per capita by age group in 2015 [18]. This figure is based on the total fee of both medical and dental public insurance schemes, and excludes the patients’ private contribution fees. According to the Survey on Economic Conditions in Health Care in 2015 [19], the proportion of dental expenses provided by the public health insurance scheme is about 85.8% of total dental health expenditure. The proportion of medical expenses borne by private fees was only 1.2% in 2015. So this figure can explain the general outline of Japanese health expenditure between the medical and dental components of the insurance scheme. Personal contributions for dental services are far higher than for medical care.

Total health expenditure per capita is 333,300 yen (3030 US$), and dental expenditure per capita is 22,300 yen (203 US$). Dental expenditure occupies 6.7% of total expenditure in general. It is amazing that those aged 65 years and older use 60% of the total health expenditure. There is considerable evidence showing the relationship between oral health and general health. Effective oral health promotion programs targeting younger generations can therefore be expected to contribute to the escalation of medical health expenditure for the elderly population.

### 6.3. Emergency Oral Health Systems in Times of Disaster

As Japan has one of the highest frequencies of natural disaster in the world, it is recognized that special systems in the field of health are necessary as risk management tools [20]. In times of disaster, the ordinary health care system may not function. In March 2011, Japan experienced its strongest-ever recorded earthquake and tsunami disaster, and a nuclear power plant accident in Tohoku area. From this catastrophic experience, we realized that not only is medical support necessary for the population affected, but also that dental support is important to allow the people to maintain health and comfort in times of disaster.

The roles of dental professionals in such times should include the following:

For victims: Identification of victims at the request of police

For survivors: Provision of emergency dental treatment

       Oral healthcare for vulnerable people (especially older citizens)

       Oral health education and oral health promotion materials

In the first stage of disaster, the first dental assessment at a shelter house was conducted by non-dental personnel. Based on their assessment for the need of dental services, dental professionals were sent to the affected area to deliver adequate dental care. An example of dental assessment items at an emergency situation was developed and is shown in Table 4.

To make sure every type of dental personnel could respond appropriately to such an emergency situation, training programs are being provided for the members of Japan Dental Association and Japan Dental Hygienists’ Association. Disaster dentistry is now included in the undergraduate dental curriculum in Japan.

### 6.4. Tele-Dental Systems in the Remote Areas without Dental Professionals

At present, there are six astronauts working in the International Space Station (ISS). These members are special crews trained to live in the space environment with no access to a dental facility. But in the near future, space technology will develop so ordinary people will also have the chance to travel or live in space.

For the purpose of the astronauts’ oral health promotion, the Faculty of Dentistry, Tokyo Medical and Dental University (TMDU) and the Japan Aerospace Exploration Agency (JAXA) cooperated to develop the “Space Oral Health Promotion Project” to tackle the current and the possible dental or oral problems in future long-term space flight [21]. It is a new challenge for us to develop “space dentistry”.

In Antarctica, Japan has the Showa Station. The Japanese Antarctic Research Expedition (JARE) team has been engaging in research for more than one year in circumstances without access to a dentist. At present, TMDU conducts tele-dental conferences with doctors in the Antarctica for dental support of JARE members. Real-time diagnosis and adequate advice for dental troubles of JARE members can be provided using an intraoral camera and a TV system. 

We believe that the tele-dental system could be expanded for other remote or rural areas with limited or no access to dental professionals. In such situations, oral self-care and prevention of dental diseases are the most important strategies. By giving adequate advice using recent advanced technologies, dentists can help these isolated population groups. Dentistry in the future may contribute to oral health promotion for people everywhere on earth and also in space.

## 7. Conclusions

Japan has developed a system for providing high-quality and appropriate oral health care efficiently. Therefore, the oral health status of the Japanese population has improved markedly. Dental caries in children decreased remarkably. In adults and older populations, untreated decayed teeth decreased and people are keeping more natural teeth than ever before.

Many factors are thought to contribute to these changes. Public oral health services are provided according to the life stage of their populations and these services are mainly conducted by private dental practitioners under contracts with local governments. The number of dental facilities increased and the health insurance system helps by providing easy access to receiving dental treatment at reasonable price. Fluoride usage has increased, and sugar consumption has decreased. People’s awareness and behavior toward oral health have also improved. Japanese dentistry is now challenging to solve the newly emerged oral health problems.

## Figures and Tables

**Table 1:** Numbers of dentists in Japan (2016).

Practicing Places	Number	%
**Dental practice**	**101,551**	**97.1%**
Private office (employer)	(59,482)	(56.9%)
Private office (employed)	(29,684)	(28.4%)
Hospital	(3,077)	(2.9%)
Education institute	(9,308)	(8.9%)
**Research institute**	**1195**	**1.2%**
**Administration/public service**	**348**	**0.3%**
**Others**	**1430**	**1.4%**
**Total**	**104,533**	**100.0%**

**Table 2:** Health related law and acts in Japan.

Law/Act	Main Target Population
Maternal and Child Health Act (1965)	Infants, preschool children, pregnant women
School Health and Safety Act (1958)	Schoolchildren
Industrial Safety and Health Act (1972)	Workers
Act on Securing Medical Care for Elderly People (2008)	Elderly
Community Health Act (1947)	All population
Health Promotion Law (2003)	All population
Act on the Promotion of Dental and Oral Health (2011)	All population

**Table 3:** Goals related to oral health in the second “Healthy Japan 21”.

Indicators	Baseline Data	Goals
**1. Maintenance and improvement of oral function**		
Increase in proportion of persons aged 60–69 years with good mastication function	73.4% (2009)	80% (2022)
**2. Prevention of tooth loss**		
A. Increase in the proportion of 80-year-old persons with 20 or more teeth	25% (2005)	50% (2022)
B. Increase in the proportion of 60-year-old persons with 24 or more teeth	60.2% (2005)	70% (2022)
C. Increase in the proportion of 40-year-old persons with no missing teeth	54.1% (2005)	75% (2022)
**3. Prevention of periodontal disease**		
A. Decrease in the proportion of persons in their 20s with gingivitis	31.7% (2009)	25% (2022)
B. Decrease in the proportion of persons in their 40s with progressive periodontitis	37.3% (2005)	25% (2022)
C. Decrease in the proportion of persons in their 60s with progressive periodontitis	54.7% (2005)	45% (2022)
**4. Prevention of dental caries**		
A. Increase in the number of prefectures where >80% of 3-year-old children are caries free	6 prefectures (2009)	23 prefectures (2022)
B. Increase in the number of prefectures where 12-year-old children have fewer than 1 DMFT (decayed, missing and filled permanent teeth)	7 prefectures (2011)	28 prefectures (2022)
**5. Regular dental check-up**		
Increase in the proportion of persons who received a dental check-up during the past year	34.1% (2009)	65% (2022)

**Table 4:** Dental checklist items for the people at a shelter house in times of disaster.

Dental Checklist Items		Contents
**D**	**D**ental high-risk population	To know the number of the dental high-risk population is important in the affected area. In the shelter house, to check the number of elderly, disabled persons, and pre-schoolchildren and to report them to the emergency disaster office is a high priority. Then the manager of the office can ask for dental support from an unaffected area.
**E**	**E**nvironmental settings	To keep good oral health, it is necessary to check the availability of water and water-supply facilities, not only for drinking but also for mouth-rinsing.
**N**	**N**ecessary support for oral hygiene behavior	Check people’s oral hygiene behavior (brushing). Can they brush by themselves or do they need special care to clean their teeth and mouth?
**T**	**T**ool materials for oral hygiene behavior	Are there enough oral hygiene tool materials in the shelter house? (e.g., toothbrush, toothpaste, dental floss, interdental brush, mouth wash, denture cleaning tablets etc.)
**A**	**A**cute dental treatment needs	Do they need acute dental treatment? Is emergency dental treatment necessary, such as acute pain and loss of dentures?
**L**	**L**imitation: Obstacles to receiving dental treatment in the affected area	How much damage to dental facilities (i.e., clinics and hospitals) is there in the affected area? Is a mobile dental service necessary?
